# The impact of online games on creativity and the role of imagination

**DOI:** 10.3389/fnbeh.2025.1561548

**Published:** 2025-05-22

**Authors:** Yung-hsun Cheng

**Affiliations:** Department of Product and Game Design, Chienkuo Technology University, Changhua City, Taiwan

**Keywords:** creativity, electroencephalogram, imagination, online games, motivation

## Abstract

**Background study:**

The rapid growth of online gaming has sparked interest in its potential effects on cognitive abilities such as creativity. Previous research indicates that interactive digital environments may enhance creative skills through immersive gameplay. However, the role of imagination as a mediator in the relationship between gaming and creativity remains under-explored. This study investigates how playing online games influences creativity and examines the mediating effect of imagination in this relationship.

**Objectives:**

The primary objectives of this research are to: (1) evaluate the direct impact of online gaming: investigate how participation in online gaming affects players’ imagination and creativity levels, measuring specific outcomes through qualitative and quantitative assessments. (2) explore the mediating role of imagination: analyze the relationship between players’ motivation for gaming and their creative output, focusing on how imagination serves as a mediating factor in this dynamic. (3) differentiate game types and their effects: assess how various genres of online games, specifically role-playing games (RPGs) and sandbox games, uniquely influence players’ imaginative capabilities and creative expressions, using comparative analyses to highlight differences in outcomes.

**Methods:**

This study employed a mixed-method approach, combining survey questionnaires and experimental procedures. The survey collected responses from 202 participants, gathering insights into their gaming behaviors, motivations, and perceived creativity. For the experimental segment, 12 participants (6 males, 6 females) were divided equally into experimental and control groups. Participants were assigned to either play *Genshin Impact* (a RPG) or *Roblox* (a sandbox game) to observe differences in creativity and imagination lasting for four weeks. Data were analyzed to determine the relationship between gaming motivation, imagination, and creativity. However, the goal of this work was to investigate whether exclusive play of either Roblox or Genshin Impact over a four-week period would differentially influence player motivation, creativity, and imagination.

**Results:**

Results from the survey and experimental data indicate a significant relationship between players’ motivation and post-game imagination. Notably, gaming motivation also had a significant impact on creativity, particularly in *Genshin Impact* and *Roblox*. The findings confirmed that imagination acts as a mediator between motivation and creativity, suggesting that players who are more motivated display enhanced creative abilities, partly due to increased imaginative engagement during gameplay.

**Conclusion:**

The study concludes that online gaming can positively influence creativity, especially when imagination mediates the relationship between gaming motivation and creative outcomes. These results underscore the potential cognitive benefits of online games, particularly for players with high levels of motivation, and highlight the distinct roles of RPGs and sandbox games in fostering creativity.

## Introduction

There is a widespread consensus in society regarding the significant impact of internet technology. Playing online games, in particular, has been frequently associated with negative consequences such as gaming addiction and disordered gaming, which can adversely affect reading comprehension and increase reliance on visual media for information (Cheng, 2021; Park et al., 2016; Pontes et al., 2014). However, research in this domain has primarily focused on the visual–spatial cognitive effects and problem-solving advantages provided by online games, often overlooking their potential to enhance imagination and creativity (Adachi and Willoughby, 2013; McGonigal, 2016; Runco, 2014).

Interestingly, certain types of video games have been noted to promote creativity. For instance, research indicates that children who engage more frequently in video gaming, including games with violent content, tend to achieve higher scores on creativity tests compared to their peers who game less (McGonigal, 2016). Further supporting this, studies conducted by Ott and Pozzi (2012) confirmed that online games can nurture creativity, leading to significant improvements in students’ creative skills and attitudes (Ott and Pozzi, 2012). Additionally, Blanco-Herrera et al. (2019) provided empirical evidence that reinforces the notion that online games hold substantial potential for enhancing creativity (Blanco-Herrera et al., 2019).

Conversely, Rafner et al. (2021) pointed out a disconnect between the potential of using online games for creativity assessment and the breadth of actual research coverage. They suggested exploring complementary approaches to design creative assessment games (Rafner et al., 2021).

Imagination, recognized as a precursor to creativity (Karwowski and Soszynski, 2008), may act as a mediating force in stimulating creativity during online gaming, necessitating further investigation in this area. Recent studies highlight that engaging in online games can trigger imagination, as indicated by increased activity in specific brain regions associated with delta and theta brainwaves (Cheng, 2021; Liang and Chen, 2013; Liang et al., 2013). However, the direct and indirect roles of “imagination” within the relationship between online gaming and creativity remain ambiguous, revealing a notable research gap.

To address this gap, a comprehensive research framework is essential for exploring the effects of online gaming on both imagination and creativity. This study aims to determine whether online games indeed contribute to the stimulation of creativity and to elucidate the nature of their direct and indirect effects, thereby highlighting the crucial roles of imagination.

## Literature study

### Motivation for playing online games

The primary motivations for playing online games include leisure, social needs, and stress relief, which drive players to engage in gaming activities. McCrae and Costa’s Five-Factor Model of personality—Openness, Conscientiousness, Extraversion, Agreeableness, and Neuroticism—has been shown to influence individual behavior and psychological development over time. Among these traits, Openness has a strong positive correlation with creativity, as individuals with high Openness tend to be more imaginative and exploratory (McCrae and Costa, 1997).

Yee (2006) conducted a study on the motivations for online gaming, identifying multiple reasons why players engage in games, including social interaction, achievement, immersion, and escapism. The diversity of these motivations helps researchers and game developers understand player needs and design more engaging experiences. Furthermore, Green and Bavelier (2003) investigated the effects of action video games on visual selective attention, finding that players of such games exhibit superior visual information processing abilities, suggesting that gaming can offer cognitive benefits beyond entertainment. The intersection of Yee’s exploration of player motivations with Green and Bavelier’s work on cognitive benefits underscores the complex relationship between gaming experiences and player development. Recognizing how different motivations influence gameplay can inform the creation of games that not only entertain but also promote cognitive skills. For instance, games designed to enhance social interaction may foster collaboration and communication skills, while those focused on achievement might encourage problem-solving and critical thinking.

Overall, both studies emphasize the need for a holistic approach to game design that aligns with player psychology and developmental objectives. By integrating insights on player motivations with findings on cognitive benefits, game developers can create experiences that are not only enjoyable but also contribute positively to players’ cognitive and social development. This approach underscores the potential of video games as valuable tools in today’s digital landscape.

Additionally, Korgaonkar and Wolin (1999) identified four primary types of motivations for internet use: social escapism, information acquisition, interactive control, and socialization needs. While these motivations share similarities with engagement behaviors in gaming, it is important to note that this categorization is not universally accepted and should be viewed as a framework for understanding rather than as established fact.

Moreover, game motivations can be classified into four main types. *Implicit motivations and biological drives* stem from innate instincts such as power, achievement, and affiliation, influencing dominant behaviors and social interactions in games. *Environmentally shaped motivations* arise from external factors like rewards and punishments, reinforcing behaviors through uncertainty-driven engagement. *Intrinsic motivations and cognitive needs* align with self-determination and flow theories, where players seek competence, autonomy, and immersion in goal-oriented gameplay. Lastly, *personality-based motivations* reflect individual differences in traits and preferences, though no single theory fully explains the link between personality and gaming behavior (Hodent, 2017a, 2017b).

For example, *implicit motivations and biological drives* stem from innate instincts such as power, achievement, and affiliation, which influence dominant behaviors and social interactions in games (Ryan and Deci, 2000). *Environmentally shaped motivations* arise from external factors like rewards and punishments, reinforcing behavior through uncertainty-driven engagement. *Intrinsic motivations and cognitive needs* align with self-determination and flow theories, where players seek competence, autonomy, and immersion in goal-oriented gameplay (Csikszentmihalyi, 1990; Deci and Ryan, 2000). These intrinsic motivations, such as challenge and autonomy, are particularly important because they can promote players’ creative development. When players are motivated by challenges, they are more likely to explore innovative strategies and solutions, enhancing their creative expression.

Additionally, different types of games, such as open-world games versus linear games, may attract specific types of players, consequently influencing their creative performance (Nacke et al., 2014). Open-world games often provide freedom and flexibility, encouraging players to experiment and express creativity, whereas linear games might limit such opportunities. This distinction underscores the importance of game design in facilitating or hindering creative engagement.

Lastly, personality-based motivations reflect individual differences in traits and preferences, although no single theory currently fully explains the link between personality and gaming behavior (Hodent, 2017a, 2017b). The motivations players experience during gameplay may also influence their creative applications both in-game and out of game. Understanding how various motivations affect players’ creativity can provide valuable insights for enhancing game design and player experiences, ultimately reinforcing the theoretical foundations of this study, and making the literature review more comprehensive.

To deepen the discussion, it is crucial to explore how various motivations can influence player creativity. For instance, certain intrinsic motivations may enhance creative expression, while different game types might attract diverse player profiles and affect their existing skills. Furthermore, if this study does not address issues surrounding gaming addiction or violence, I explicitly clarify that these topics are outside the scope of my research to prevent any misunderstandings.

To further supplement this topic, Di Bello et al. (2019) explored how incentive motivation affects attentional focus. They suggest that when subjects are driven by strong external rewards, such as achievements or prizes obtained within a game, their attention becomes more concentrated on stimuli associated with those rewards. This implies that the incorporation of incentives and rewards in game design not only enhances player engagement but also cognitively strengthens their processing of important information during gameplay. In addition, Engelmann and Pessoa (2007) investigated the concept of motivational modulation of attention. They found that motivation can significantly enhance the effects of exogenous attention, particularly in the context of reward acquisition. This indicates that when players see highly motivating rewards in a game, their visual systems naturally focus more on stimuli related to those rewards, thereby enhancing both the interactivity and enjoyment of the game. Finally, Chelazzi et al. (2013) further examined how rewards teach selective visual attention. Their experiments demonstrated that rewarded stimuli can guide players’ attention, making their responses to relevant information faster and more accurate. This finding suggests that cleverly arranging reward mechanisms in game design can help players concentrate on the most challenging aspects of the game, while also improving their overall gaming performance.

### The relationship between player motivation and creativity

Research shows that player motivation plays a crucial role in enhancing creativity. Intrinsic motivations, such as challenge and autonomy, often stimulate players’ creative thinking because these motivations encourage players to explore new ideas and solutions (Deci and Ryan, 2000). For example, open-world games typically provide greater freedom and flexibility, attracting players who wish to express their creativity (Gee, 2007). By emphasizing the role of challenge and autonomy, it becomes evident that games designed to promote these elements can significantly enhance players’ creative output. Open-world games exemplify this well, as they allow players the freedom to explore and create, indicating that game design principles can effectively tap into players’ intrinsic motivations.

Additionally, different types of games can appeal to different types of players, thereby enhancing their existing skills during gameplay (Amabile, 1996). This suggests that game design can effectively guide players to leverage their interests and motivations to promote creative development (Csikszentmihalyi, 1996). Research also indicates that social motivations can strengthen creativity, as players often gain inspiration and new perspectives through collaboration and social interaction (Runco and Acar, 2012). By tailoring game experiences to these different motivations, developers can enhance skill development and promote creativity. The integration of social motivations further enhances this potential, as collaborative experiences can lead to richer idea generation and innovation. This insight emphasizes the importance of multiplayer dynamics and community building in fostering a creative gaming environment.

In summary, online gaming motivations encompass various psychological and social factors, ranging from escaping academic pressure to fulfilling social needs. These motivations not only influence gaming habits but also contribute to immersion and, in some cases, gaming addiction. Therefore, further exploration of gaming motivations and their impact on players’ psychology and behavior is essential for understanding how online gaming affects cognitive development and creativity.

### Rationales of imagination

Imagination is a critical aspect of creative intelligence, enabling exploration of alternative solutions and visualization of possibilities. Imagination and creativity are linked to intention, deviance from rules, and personality traits, including possibility thinking. Imagination precedes creativity and involves conceiving and transforming ideas into plans, while creativity involves taking action based on imagination (Karwowski and Soszynski, 2008; Lin et al., 2014; Seligman and Kaufman, 2014). Psychological factors, including intrinsic motivation, self-efficacy, stress, action inspiration, emotion, and generative cognition, are stimulated by imagination, with generative cognition acting as a mediator (Hsu et al., 2014). Imagination can be categorized into two dimensions: creative imagination, which emphasizes new ideas and originality, and reconstructive imagination, involving the reconstruction of existing mental images (Colello, 2007). Wang et al. (2014) propose that imagination involves generating many ideas, linking relationships between ideas, restructuring and separating relationships between ideas, and selecting materials for design drawings (Wang et al., 2014). Imagination serves as a powerful tool for innovation and can be applied extensively in various fields, including anthropology (Hayes et al., 2017). Cheng (2021) found that creative construction games effectively stimulate imagination compared to team action-adventure games (Cheng, 2021). However, they did not significantly improve the generation of high-quality images like originality. The cognitive load theory, combined with an evolutionary perspective, suggests that students with sufficient prior knowledge benefit from imagination (Wang et al., 2022). Imagination plays an inspiring role in cognitive learning, influencing the generation of creativity directly or indirectly.

### Rationales of creativity

Regarding creativity, it can be classified into normative creativity, which involves problem-solving for established tasks, and exploratory creativity, used to explore future possibilities (Winkelhofer, 2006). Creativity tests, such as Torrance’s test of creative thinking, measure divergent thinking abilities, including fluency, flexibility, originality, and elaboration. The Torrance Tests of Creative Thinking (TTCT) is a widely used tool for assessing individual creative thinking. It is broadly used in education and psychology to help identify creative talent (Almeida et al., 2008). Csikszentmihalyi’s theory emphasizes that creativity emerges from the interaction between individuals, products, and the environment (Csikszentmihalyi, 1999). The creative process involves innovative behavior, astonishing techniques, and continuous changes in cognition. In the context of art, creativity redefines beauty and transforms known elements (Beattie, 2000). Cognitive retrieval investment and variation play a role in creative performance in the field of art (Acar and van den Ende, 2016). Online games that feature ill-structured or open-ended challenges have the potential to enhance intrinsic motivation and facilitate flow experiences, thereby supporting the development of creativity (Hall et al., 2022; Kiili, 2005). The “Creativity in Gaming Scale (CGS)” by Hall et al. (2022) measures the effectiveness of games, particularly in promoting player creativity. It helps learners and teachers track and reflect on creativity development over time (Hall et al., 2022). The scale consists of five dimensions: transferability, appropriation, problem-solving, affective change, and design affordances. It comprises 22 items. In summary, the CGS is primarily designed to assess players’ tendencies to engage in creativity-oriented gameplay and to evaluate the extent to which games facilitate creativity. The scale encompasses not only creative behaviors such as strategy development and character customization, but also the broader impact of gaming on players’ attitudes, behaviors, and self-perceptions in everyday life. As noted by Hall et al. (2022), the CGS serves as a valuable tool for identifying which aspects of creativity are relevant to specific games, game genres, or player demographics, and holds potential for tracking creativity development over time.

### Causal relationships and mediation effect between variables

Regarding the causal relationships and mediation effects of imagination, Liang et al. (2013) studied design students and found that imagination mediated the relationship between personality traits and creativity (Liang and Chen, 2013). Imagination also played a mediating role between creativity and academic performance, with certain aspects of imagination positively influencing academic performance (Lin et al., 2014). Personality traits like openness, conscientiousness, and agreeableness predicted original or useful creativity through imagination (Hsu, 2019). Moreover, creative imagination and novelty of thinking mediated the improvement of creativity (Groyecka et al., 2020). These findings suggest that playing online games can predict imagination and mediate its influence on creativity, supporting research hypotheses H1 and H3.

Moreover, regarding causal research on online games and creativity, Ott and Pozzi (2012) observed significant improvements in cognitive abilities and technological creativity in students exposed to online games (Ott and Pozzi, 2012). Integrating online games into the curriculum can enhance students’ creativity (Lin et al., 2011), supporting research hypothesis H2.

## Methods

### Research framework and hypotheses

#### Research framework

**Figure 1:**
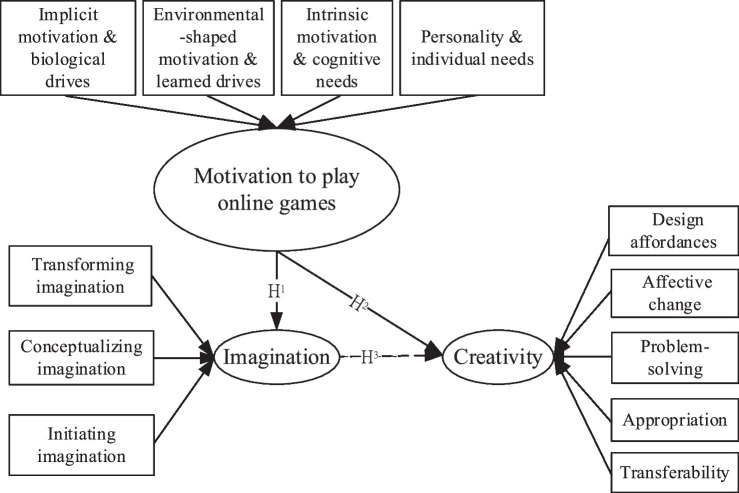
Research framework.

The study’s research framework includes participants playing online games as the independent variable. They undergo surveys on imagination and creativity, as well as pre-test and post-test of brainwave EEG. Direct effects are represented by solid lines, and indirect effects by dashed lines, as shown in Figure 1.

#### Research Hypotheses

The study hypothesizes that the “motivation to play online games” predicts “imagination” and “creativity,” with “imagination” acting as a mediator. Moreover, if the motivation to play online games is positively related to creativity, with imagination serving as a mediating factor in this relationship. Specifically, it is posited that increased motivation to play online games enhances imaginative thinking, which in turn fosters greater creativity. This hypothesis emphasizes the role of imagination as a crucial intermediary that influences how gaming motivation translates into creative outcomes. The specific research hypotheses are as follows (also see Table 1):

**Table 1:** Experimental design procedure.

Group	EEG pre-test	E: Genshin impact C: Roblox	EEG post-test
Experimental Group	R E^1^	X^1^	E^2^
Control Group	R C^1^	X^2^	C^2^

R: Random sampling; X: Treatment; C: Experimental control. E^1^-E^2^ and C^1^-C^2^: EEG Pretest and Post-test (Cognionics Quick-20 and NuroStat software). X:"Genshin Impact” has the highest online buzz volume (166,893) (Play Tracker, 2020). C: “Roblox” has the highest positive influence rating (8.08) (Statista, 2024a, 2024b). Source: Created by the author.

*H1*: The motivation to play online games, specifically Genshin Impact or Roblox, is positively associated with levels of imagination.

*H2*: The motivation to engage in Genshin Impact or Roblox online games is positively associated with creativity.

*H3*: The motivation to play online games is positively related to creativity, with imagination serving as a mediating factor in this relationship.

### Objective

This study focuses on Taiwanese university students as the research subjects. Purposive sampling was used to select students from various colleges, departments, academic years, and genders, aiming for proportional representation. Data collection was conducted through online questionnaires and scales (Google Forms), with four main variables and a total of 56 items. It was expected to collect 202 valid samples (freshmen: 62 students, sophomores: 51 students, juniors: 47 students, seniors, and extended students (all students who have exceeded the general four-year learning period.): 42 students; accounting for 30.69, 25.24, 23.27, and 20.79%, respectively.). Moreover, to enhance the validity and reliability of this study, an experimental research method was employed, and purposive sampling was used to select 6 male and 6 female participants. The sampling criteria are college students, and participants must have a basic understanding and experience of the selected games (such as Genshin Impact or Roblox). For the EEG experiment, participants should be in good health and free from conditions that could affect EEG readings (such as epilepsy or other neurological disorders) and must participate voluntarily. Moreover, they were randomly assigned, with an equal number of males and females in each of the experimental and control group, totaling 12 participants. The two groups played different types of online games, while the all participants are prohibited from playing other online games during the experiment to avoid affecting the accuracy of the research. The specific control measures as follows:

Dedicated Experimental Platform: A dedicated gaming platform was provided in a controlled environment, which was locked to only allow the games related to the experiment.

Self-Reporting: Participants, being volunteers who understood the purpose of the experiment, were able to self-regulate. Additionally, participants were instructed not to engage in additional gaming activities during the study period.

Control of the Experimental Environment: The experiment was conducted in a laboratory where researchers could control the entire environment, ensuring that participants could not access other games or websites.

Interference Control: Participants were instructed after completing the experiment not to engage in any other gaming activities for the remainder of the data collection period, in order to minimize potential external influences on the measured outcomes.

Through these measures, the internal validity of the experiment was strengthened, ensuring that the data collected was accurate.

The experimental treatment (playing online games) for the groups was conducted twice a week, with each session lasting 2.5 h, over a period of 4 weeks, totaling up to 20 h. Additionally, the pre-test and post-test of brainwave EEG (electroencephalography) for each participant took approximately 30–40 min. Brainwave patterns were collected and interpreted to determine changes in brain regions, brainwave frequencies, and participants’ emotional variations, ensuring the accuracy of the research findings. The entire process took approximately 2 h, including the explanation of the EEG data collection method, participants’ sitting time, and preparation time. The experimental procedure is presented in Table 1. To make the objectives of the experiment clearer, this study presented the following experimental hypotheses:

Experimental Hypotheses:

*H1*: The motivation to play online games such as Genshin Impact or Roblox positively predicts players' levels of imagination.

*H2*: The motivation to play online games such as Genshin Impact or Roblox positively predicts players' levels of creativity.

*H3*: The motivation to play online games positively predicts creativity, with imagination serving as a mediating factor in this relationship.

These hypotheses specify the relationships between motivation, imagination, and creativity, establishing a foundation for the research analysis.

### Tools

#### Selection of online games

The reason for choosing these two games in this study is based on the following considerations: First, *Roblox* and *Genshin Impact* represent two distinctly different gaming experiences—*Roblox* emphasizes open creation and user-generated content (UGC), while *Genshin Impact* focuses on immersive storytelling and role-playing. This study aims to explore the impact of different types of games on player creativity; therefore, selecting these two games allows for a comparison that analyses how open creative environments versus predefined narrative environments influence the development of player creativity. Additionally, *Roblox* is set as the control group due to previous research investigating the relationship between open-world creative games and creativity. For example, existing studies have pointed out that free construction and creative gaming environments can promote divergent thinking in players (Deterding, 2015). By using *Roblox* as a reference, it becomes possible to more clearly examine whether games like *Genshin Impact*, which have predefined worlds, can also stimulate players’ creative thinking or if their influencing mechanisms differ.

##### Genshin impact

*Genshin Impact* is an open-world adventure role-playing game (RPG) set in the fantasy world of “Teyvat.” In this RPG, players can unlock over 30 characters by completing quests and utilizing a loot-box gambling system for drops through money or in-game resources. Once players reach Adventure Rank 16, they can also engage in co-op gameplay, where up to four players can team up and play together (miHoYo, 2024).

##### Roblox

“*Roblox* (sandbox game)” allows both aspiring game creators to focus on realizing their game creation dreams and helps more game developers achieve rapid monetization, turning dreams into reality (Blake and Barnes, 2023). Official data reveals that Roblox has over 50 million daily active players in 180 countries. Players can develop their own games, interact with different avatars, resulting in a countless number of games, and a wide variety of genres. It has been described as the initial phase of the metaverse by the industry (Roblox, 2022). In summary, *Roblox* has the highest positive influence rating and is representative, making it suitable for inclusion in this study’s experimental treatments.

#### Online games motivation scale

This study’s *Online Gaming Motivation Scale* is structured based on the motivational theory proposed by Hodent (2017a, 2017b), and also draws on the *Pokémon GO Gaming Motivation Questionnaire* used by Cheng (2019) as well as the motivational scale developed by Korgaonkar and Wolin (1999). The scale’s validity and reliability were evaluated using the following statistical measures: the Kaiser-Meyer-Olkin (KMO) measure of sampling adequacy was 0.907, and Bartlett’s Test of Sphericity yielded a chi-square approximation of 1330.019 with 120 degrees of freedom and a significance level of 0.000. The reliability coefficient, Cronbach’s *α*, was calculated at 0.936. Additionally, the factor loadings for each item ranged from 0.793 to 0.530. Moreover, the total variance explained reached 69.365%, indicating a strong explanatory power of the scale. These findings demonstrate that the *Online Gaming Motivation Scale* is both valid and reliable.

#### Imaginative capability scale

In terms of imaginative capability, the 29-item Imaginative Capability Scale developed by Liang and Chia (2014) was used for assessment (Liang and Chia, 2014) in the study. Participants self-evaluated their level of agreement on the dimensions of initiating imagination, conceptualizing imagination, and transforming imagination using a six-point scale. The confirmatory factor analysis of the scale showed good fit indices: *χ^2^* (374) = 1588.81, *p* < 0.05, CFI = 0.96, RMSEA = 0.08, SRMR = 0.07, NFI = 0.95, NNFI = 0.96, indicating a good fit of the sample model. The standardized parameter estimates of the remaining imaginative capability items ranged from 0.51 to 0.80 (Liang and Chia, 2014), meeting the recommended standards for examination by Hair et al. (2014). Additionally, the composite reliability for initiating imagination was 0.90, for conceptualizing imagination was 0.92, and for transforming imagination was 0.89. Overall, the imaginative capability scale exhibited strong internal consistency (Fornell and Larcker, 1981). Liang and Chia (2014) proposed a scale comprising 29 items, which is a well-designed scale. However, due to the large number of items, to prevent respondents from becoming bored or fatigued, which could affect the sincerity of their answers and thus the scale’s reliability and validity, the authors selected representative items from questions with similar meanings. Each dimension contains 4 items, totaling 12 items, including items originally numbered 1, 4, 6, 7, 11, 14, 15, 19, 23, 25, 26, and 29.

#### Creativity capability scale

The Hall et al. (2022) Online Games Player Creativity Scale consists of 26 items. The measure Creative Ability Scale (Hall et al., 2022) used in this study has demonstrated a high degree of construct validity and convergent validity for the 5-factor model, comprising Transferability, Appropriation, Problem-Solving, Affective Change, and Design Affordances. This model underwent the same iterative process as the original factor model. All items with loadings below 0.45 and those with cross-loadings were removed, and Principal Component Analysis (PCA) was rerun until no items remained with cross-loadings or loadings below 0.45. The final model accounted for 57.4% of the variance. The Kaiser-Meyer-Olkin measure of sampling adequacy was good at 0.835 and Barlett’s Test of Sphericity was significant (*X^2^* = 2303.942, *df* = 325, *p* = 0.000). Determinant was above the acceptable 0.0001. The instrument was named the Creativity in Gaming Scale (CGS) (Hall et al., 2022). This scale is well-constructed; however, it comprises an excessive number of items. To mitigate the risk of respondents experiencing boredom or fatigue—factors that can compromise the sincerity of their responses and ultimately affect the scale’s reliability and validity—the author has decided to remove items with factor loadings below 0.7. The guiding principle is to limit each dimension to no more than five items. Consequently, the items designated for removal are 26.2, 25.3, 27.10, and 24.1 form the original version, resulting in a total of 22 items retained in the scale.

#### Wireless EEG headset

The Quick-20 dry electrode EEG headset is the latest mobile EEG technology that provides usability and signal quality to support new research applications. The Cognionics Quick-20 is a wireless electroencephalogram (EEG) head-mounted device that offers several advantages, including wireless connectivity, portability, and comfort. It allows for multi-channel measurements, providing more comprehensive EEG data with high quality. The device can capture subtle brainwave activities while suppressing external interference. All participants in the study will undergo a pre-test analysis of brainwave data recorded using the EEG cortical layer. At the conclusion of the experiment, all participants will undergo a post-test where the average and standard deviation of brainwave frequencies will be collected. Below are the specific descriptions of data collection and processing for the Quick-20 dry electrode EEG headset (NuroStat Tutorial and Manual Version 2.0, n.d.):

#### Data collection conditions

##### Sampling frequency

The Quick-20 EEG headset typically has a standard sampling frequency, such as 250 Hz or 500 Hz, depending on specific application needs. Selecting an appropriate sampling frequency allows for capturing enough detail of brain wave activity, considering the specific scope of the research.

##### Electrode layout

The Quick-20 headset is usually designed according to the International 10–20 system, which provides a standardized electrode placement that ensures comparability of data. The electrodes are distributed across important areas such as the forehead, top of the head, and sides to capture activity from different brain regions.

#### Data collection environment

##### Noise control

During data collection, it’s essential to choose a quiet environment to minimize external noise interference with brain activity. Soundproof facilities or earplugs may be used to increase environmental stability.

##### Participant preparation

Before starting data collection, participants should follow a specific preparation process, such as staying awake, avoiding caffeine intake, and familiarizing themselves with the experimental content to minimize the effects of emotional and physiological states on the data.

#### Data preprocessing steps

##### Artifact rejection

In this step, identify and remove artifact data (such as noise caused by eye movements, muscle activity, or heartbeat) through visual inspection and automated algorithms. Common artifact rejection algorithms include Independent Component Analysis (ICA) and thresholding methods.

#### Filtering methods

##### Low-pass filter

This removes signals above a certain frequency (for example, 30 Hz) to eliminate muscle noise and other high-frequency interference. This is often necessary for identifying EEG bands such as alpha, beta, and theta waves.

##### High-pass filter

Typically set between 1 Hz and 0.5 Hz to remove low-frequency artifacts, such as baseline drifts.

##### Handling missing values

When dealing with missing data, interpolation methods can be used to estimate the missing values, or in extreme cases, corresponding trials may be excluded. The method chosen should be weighed against the quality of the participant’s data and the research objectives.

**Figure 2:**
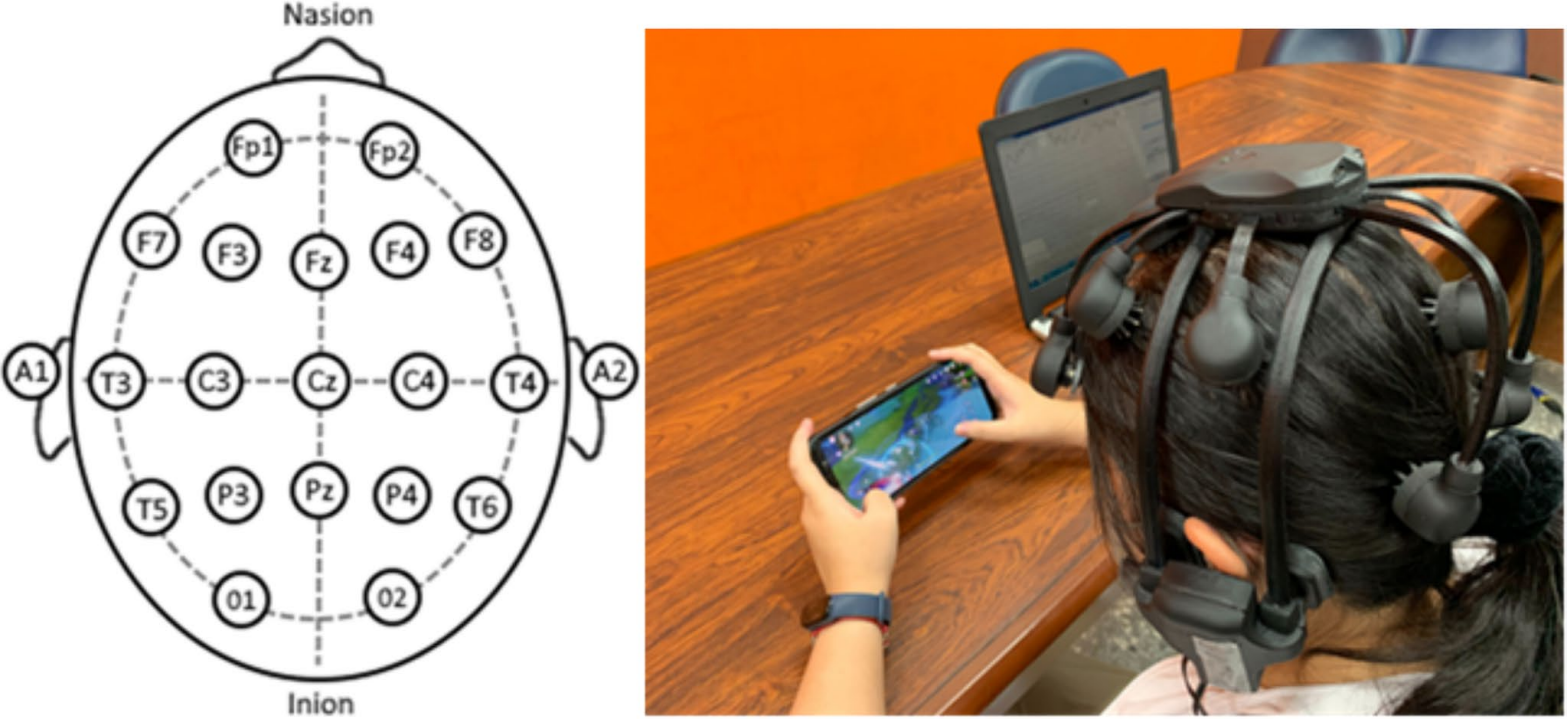
The Quick-20 dry electrode EEG headset and standard 10–20 layout. Sources: NuroStat Tutorial and Manual Version 2.0 (n.d.) and photographed by the author.

**Figure 3:**
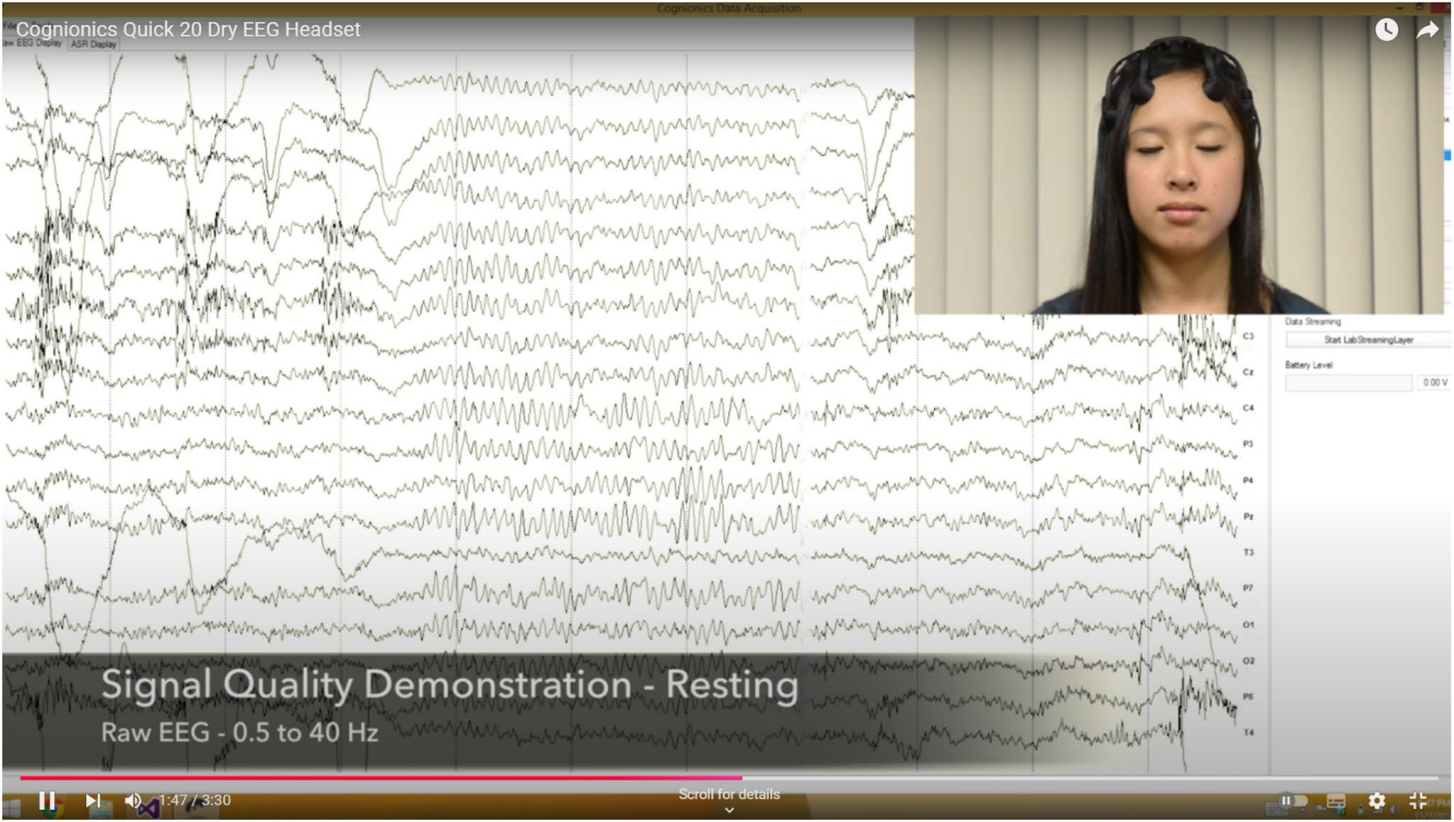
The Cognionics data acquisition software. Sources: NuroStat Tutorial and Manual Version 2.0 (n.d.).

**Figure 4:**
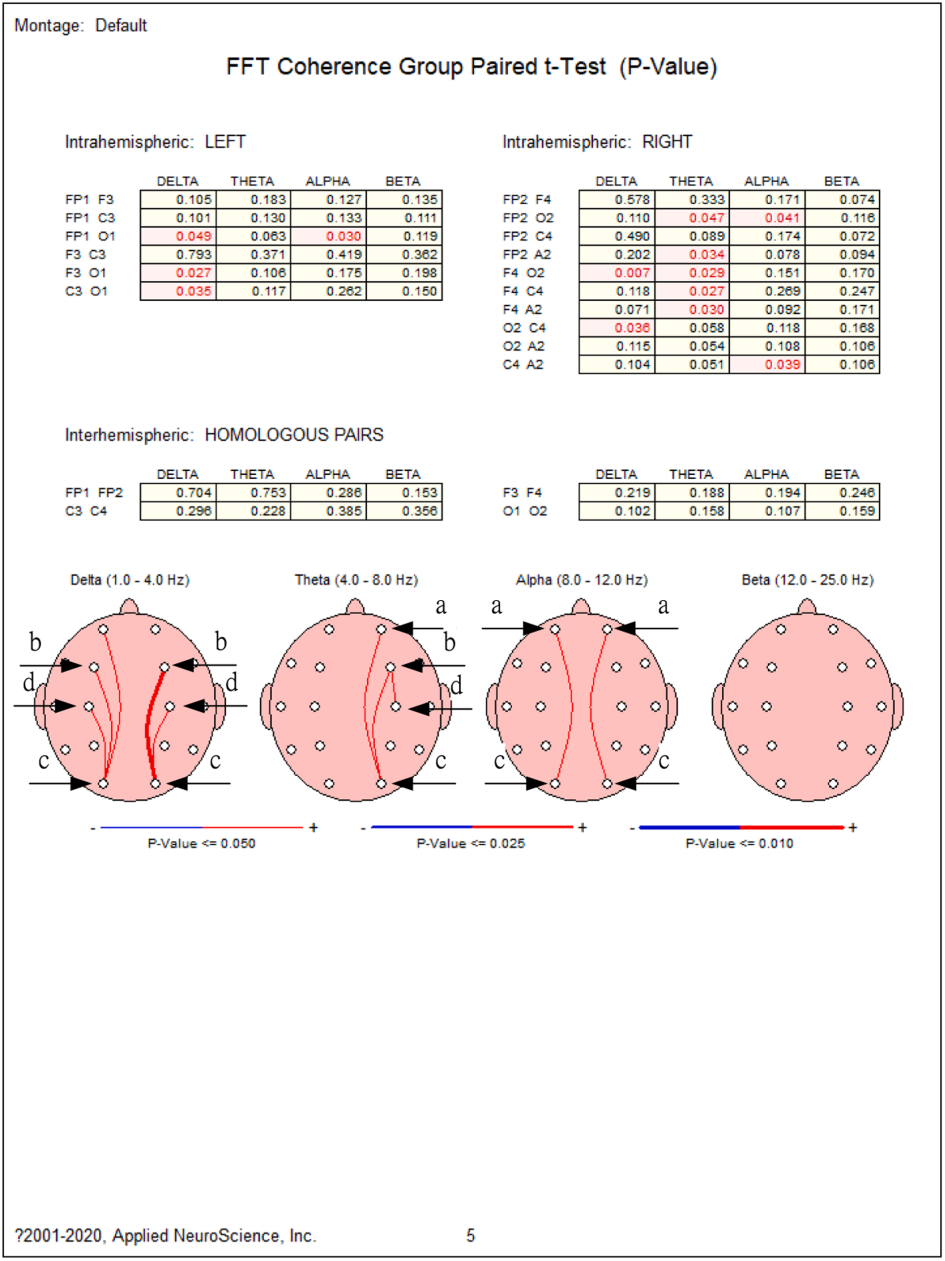
EEG analysis in paired *t*-test and maps of brain artifacts. 1. Interhemispheric left and right: The red numbers represent t-test results that are statistically significant. Also refer to Figure 2. The Quick-20 dry electrode EEG headset and standard 10–20 layout. 2. The red arc on the head represents that the posttest is larger than the pretest. The same applies in reverse. Sources: NeuroGuide Deluxe, NuroStat, 3.2.4., NeuroGuide™ by Applied Neuroscience, Inc.

These steps ensure that the collected data is appropriately processed, thereby enhancing the accuracy and reliability of subsequent analyses, also see Figures 2–4.

### Data analysis

#### Scale statistical analysis

Structural Equation Modeling (SEM) is a statistical method used to test complex statistical relationship models. AMOS is a plugin in IBM SPSS software specifically designed for conducting structural equation modeling analysis. The reasons and advantages of using SEM and AMOS software are as follows (Byrne, 2016; Hair et al., 2014; Kline, 2016).

##### Model fit testing

SEM can assess the fit between observed data and theoretical models, optimizing the model to better reflect the data.

##### Handling latent variables

SEM deals with latent variables, which cannot be directly observed but can be indirectly measured through indicator variables, providing insights into complex relationships between variables.

##### Exploration and verification

SEM combines confirmatory and exploratory analysis, allowing for the verification of previous theories or hypotheses while exploring new models or relationships.

##### Multivariate analysis

SEM addresses relationships among multiple variables, including measurement and structural models, facilitating a deeper understanding of complex interactions between variables.

##### Parameter estimation

AMOS software uses the maximum likelihood method for parameter estimation, handling complex models with numerous variables in an effective and robust manner.

##### Visualization

AMOS software provides an intuitive graphical interface for easily creating and modifying structural equation models, generating path diagrams and equation plots, making it easier for researchers to understand the model structure.

#### Testing with wireless EEG headset and NuroStat software

NuroStat is a powerful component of the NuroGuide system that is designed to facilitate the analysis and interpretation of electroencephalographic (EEG) data. Here are the primary features and functionalities of NuroStat (NuroStat Tutorial and Manual Version 2.0, n.d.):

##### Data import and preprocessing

NuroStat allows users to import raw EEG data from various recording systems. It provides tools for preprocessing, including filtering to remove noise, artifact rejection, and segmentation of the data into epochs for further analysis.

##### Statistical analysis

NuroStat offers a range of statistical tools to analyze EEG data. Users can apply various statistical tests to determine differences in brain activity across conditions or groups. This includes t-tests, ANOVA, and regression analyses, enabling researchers to assess the significance of their findings.

##### Topographic mapping

The software includes features for creating topographic maps of brain activity, allowing for visual representation of EEG data across different brain regions. This helps researchers easily identify patterns of brain activity associated with specific cognitive processes or experimental conditions.

##### Frequency band analysis

NuroStat enables the analysis of brainwave frequencies (delta, theta, alpha, beta, and gamma bands) to evaluate how different frequency bands contribute to various cognitive functions and behaviors.

##### Connectivity analysis

The software supports the examination of functional connectivity between different brain regions. This is critical for understanding how brain networks interact during specific tasks or emotional states.

##### Statistical significance mapping

NuroStat provides users with the ability to create statistical significance maps, highlighting regions of the brain that show significant differences in activity between conditions or groups.

##### Report generation

After completing the analysis, NuroStat allows users to generate comprehensive reports that include statistical results, visual representations of data, and interpretations. This functionality is vital for sharing findings with the research community or for clinical assessments.

##### Integration with other NuroGuide tools

NuroStat seamlessly integrates with other components of the NuroGuide suite, providing a holistic approach to neurophysiological data analysis. Users can easily switch between different tools for comprehensive neurofeedback training, assessment, and research purposes.

#### Analysis model

Firstly, factor loadings of all items in the three scales were greater than 0.6, except for item 1 of implicit motivation and biological drives (0.301), item 1 of environmental-shaped motivation and learned drives (0.283), and item 4 of intrinsic motivation and cognitive needs (0.545) in the motivation to play online games scale, which were deleted. Additionally, item 1 of initiating imagination (0.317) in the imaginative scale, item 1 of transferability (0.280), and item 4 of affective change (0.589) in the creative scale were removed. Furthermore, item 3 of ability to generate creative (0.276) in the creativity self-efficacy scale was also eliminated. The composite reliability in all three scales ranged from 0.789 to 0.933, all exceeding 0.7, indicating that a high proportion of the total variance in these observed indicators can be explained by the latent variable. The statistically acceptable levels of correlations supported acceptable reliability among the research constructs.

Secondly, all dimensions of the average variance extracted (AVE) in square root (0.709 ~ 909) were greater than 0.5. Most of the square root of AVE were larger than the coefficient of correlation between dimensions, except for a few dimensions such as initiating imagination vs. conceptualizing imagination, initiating imagination vs. transforming imagination, transferability vs. appropriation, appropriation vs. problem-solving, ability to generate creative vs. transferability, and ability to generate creative vs. appropriation. However, the analysis results met the criteria and indicated that the scales demonstrated discriminate validity in general. Therefore, the scales were considered acceptable in the study.

Finally, confirmatory factor analysis (CFA) was conducted. Following standard structural equation modeling (SEM) practices, path coefficient estimates and structural model-fit indices were the primary considerations (Fornell and Larcker, 1981). Using creativity as the endogenous variable and motivation to play online games and imagination as exogenous variables, each path in the model was tested. The focus was to estimate the predictive power of the independent variables on creativity, particularly the direct and indirect effects of imagination on creativity. All indices were in line with recommended benchmarks for acceptable fit (*X^2^/df* = 1.902, GFI = 0.936, AGFI = 0.894, and RMSEA = 0.067). The total explained variance in creativity was 70.942% (*R^2^* = 0.709).

## Result and discussion

### Method 1: scale survey

H1: The adjusted *R*-squares (explained variance) for Motivation in predicting Imagination for experimental group and control group are 0.629 and 0.779, respectively. The F-tests are significant, with *F*_(1, 10)_ = 19.636**, *p* = 0.001 for experimental group, and *F*_(1, 23)_ = 85.602***, *p* = 0.000 for control group. The standardized coefficients (*β*) are 0.814 for experimental group and 0.888 for control group. These results indicate that both groups treatments, representing motivations for playing different games (Genshin Impact and Roblox), have predictive power for imagination. Stronger motivations are associated with richer imagination. Despite the distinct nature of “Genshin Impact” and “Roblox”—with the former emphasizing storytelling and combat and the latter focusing on creativity and freedom—the findings suggest that motivation significantly influences post-game imagination. Players’ motivations, which may involve factors such as competition, social interaction, and creativity, are associated with more active engagement, exploration, and creative expression in the game. This higher investment of effort by highly motivated players likely stimulates richer imagination. This research highlights the significant impact of motivation on imagination after playing online games, offering practical insights for game designers and educators. Designers can enhance player motivation by incorporating appealing game elements, thereby boosting imagination. In education, leveraging the characteristics of online games can promote students’ creative thinking. Previous studies confirm that motivation is linked with enhanced imaginative thinking, particularly when players feel engaged (Deci and Ryan, 1985; Przybylski et al., 2010). In online games, both intrinsic and extrinsic motivations promote deeper engagement, leading to cognitive benefits such as imagination (Ryan and Deci, 2020). Research by Gentile et al. (2014) also suggests that immersive and interactive gaming encourages mental simulation and imagination, which aligns with this study’s findings. This study’s findings are consistent with the literature, affirming that motivation enhances imagination, regardless of game type. However, the combination of role-playing mechanics (Genshin Impact) and creative freedom (Roblox) demonstrates that different game designs can foster imagination in unique ways. This aligns with prior work showing that both structured and open-ended environments stimulate creative cognition differently (Anderson and Rainie, 2012). In summary, motivation is not only a key indicator of player retention, but also a powerful driver of creativity. If game tasks are carefully designed to trigger intrinsic motivation—such as allowing players to explore character backstories or offering freedom in mission selection—they can foster deeper psychological engagement and stimulate imaginative thinking. Moreover, in the design of educational games or learning platforms, simulating the narrative immersion found in Genshin Impact or the open-ended creative environment of Roblox can more effectively spark students’ imagination. This is especially valuable in courses involving language expression, storytelling, and design thinking.

H2: The adjusted *R*-squares (explained variance) for motivation in predicting creativity for experimental group and control group are 0.835 and 0.699, respectively. The *F*-tests are significant, with *F*_(1, 10)_ = 56.867***, *p* = 0.000 for experimental group, and *F*_(1, 23)_ = 56.630***, *p* = 0.000 for control group. The standardized coefficients (*β*) are 0.922 for experimental group and 0.843 for control group. These results indicate that both groups treatments, representing motivations for playing the two designated games (*Genshin Impact* and *Roblox*), have predictive power for creativity. Stronger motivations are associated with richer creativity. The study findings reveal that players’ motivations significantly influence their creativity in both “*Genshin Impact*” and “*Roblox*.” Stronger motivations, such as a desire to complete tasks, excel, satisfy curiosity, or engage in social interactions, lead to greater creativity. Highly motivated players are likely to invest more time and effort, exploring various possibilities and expressing creativity within the game. The games offer opportunities for creativity and freedom, with these features encouraging and stimulating players to be more creative.

Additionally, there is a significant correlation between player motivation and creativity in both “*Genshin Impact*” and “*Roblox*,” despite their differing focuses—story-driven combat versus creativity and freedom. Motivation plays a crucial moderating role in creativity, with stronger motivations leading to more abundant creative expression. These findings suggest that both games, with their expansive worlds and open environments, allow players to freely unleash their imagination and creativity. This research has practical implications for game designers and educators. Designers can enhance motivational elements within games to stimulate greater creativity in players while gaining insights into the relationship between motivation and creativity. Educators can leverage the creative aspects of these games to foster students’ creative thinking. The findings are consistent with Amabile’s (1983) theory of creativity, which highlights that intrinsic motivation is a critical factor for creative engagement. Moreover, Przybylski et al. (2010) found that motivation in digital environments leads to innovative problem-solving and artistic expression. Kafai and Burke (2015) explored how creative tasks in online games promote self-expression and social collaboration, suggesting that motivated players are more likely to engage in creative exploration. The results align well with existing literature, confirming that higher motivation leads to enhanced creativity. This study provides further evidence by showing how imagination acts as a mediator between motivation and creativity (see H3). The dual focus on structured (goal-oriented) and unstructured (free-play) games aligns with prior research, which suggests that game-based learning promotes creativity in both structured and exploratory contexts (Gee, 2003). To sum up, the connection between creativity and gaming motivation holds far-reaching implications for various industries. For instance, designers of gamified learning environments can enhance students’ creative problem-solving by increasing players’ sense of task autonomy and providing meaningful feedback mechanisms. This approach encourages learners to explore multiple pathways to solutions. Furthermore, such insights can be extended to corporate training contexts, where open-ended virtual simulations can foster creative thinking and collaborative innovation. The author could further elaborate on whether the findings of this study hold consistent across different cultural backgrounds. Social values—such as achievement orientation or collectivism—may influence the types of motivation individuals experience, which in turn could affect creative performance. This opens potential for future cross-cultural comparative research in the context of gaming motivation and creativity.

H3: The results indicate that the total effect of motivation to play online games on creativity is 1.02, comprising a direct effect of 0.77*** and an indirect effect of 0.25 (0.45*** × 0.55***), which confirms the mediating effect of imagination in the relationship between motivation and creativity. In other words, when both motivation and imagination are present, imagination enhances the positive impact of motivation on creativity. The significance of these findings lies in highlighting the crucial role of imagination in the relationship between game motivation and creativity. Strong motivation during online gaming enhances players’ engagement and active participation, while enriched imagination further facilitates creative involvement and the generation of unique ideas and solutions. The results demonstrate that motivation directly impacts creativity and also exerts an indirect effect through imagination. When motivation and imagination coexist, imagination amplifies the positive influence of motivation on creativity. This suggests that fostering and enhancing imagination can elevate players’ creativity in online games, unlocking and stimulating their intrinsic creative potential. This research underscores the importance of imagination as a key factor in the link between gaming motivation and creativity. This mediating effect is consistent with Aziz-Zadeh et al. (2013) and Fink et al. (2014), who found that imagination plays a crucial role in creative thinking processes. The idea that imaginative engagement strengthens the impact of motivation aligns with Guilford’s (1967) theory of divergent thinking (Barbot et al., 2016). Other studies, such as Plucker et al. (2004), also emphasize the importance of imagination as a precursor to creativity, especially in open-ended tasks. The mediating role of imagination confirmed by the current study extends the findings of prior research by highlighting how gaming environments can foster creative expression through imagination. This insight offers practical implications for game designers, who can focus on designing elements that stimulate both imagination and creativity to enhance player engagement. While the study clearly identifies imagination as a mediator, it would be valuable to further elaborate on its transformational role. Specifically, imagination functions as a mental simulator—translating abstract motivation into concrete creative behavior. It allows individuals to internalize goal-oriented scenarios driven by motivation and mentally rehearse possible outcomes, which in turn facilitates the generation of novel ideas. It is recommended to draw on Johnson-Laird’s Mental Models Theory (Johnson-Laird, 1983), which posits that human beings construct mental representations (i.e., imaginative models) to simulate hypothetical situations when reasoning through innovative solutions. This theory supports the view of imagination as a cognitive tool for projection, evaluation, and creative ideation. The design of games could include “imaginative triggers”—such as nonlinear storylines, multiple possible endings, or scenario-based choices—that encourage players to actively engage their imagination in predicting, exploring, and problem-solving. These features can significantly enhance creative output by prompting deeper imaginative engagement.

### Method 2: EEG analysis

H1: The paired group *t*-test *t*-values for motivation to imagination in the experimental and control group are *p* < 0.001 and *p* < 0.05, respectively. The prefrontal cortex, temporal lobe, and parietal lobe show higher activity, suggesting a notable disparity between pre-test and post-test scores of imaginations in both groups. The study shows that both groups exhibit a positive correlation in improving imagination. These results support the findings of the scale survey (H1) and enhance the overall reliability and validity of the study. These findings align with research by Ellamil et al. (2012a, 2012b) and Fink et al. (2014), who identified the prefrontal cortex and parietal lobe as critical regions for creativity and imagination. Similarly, the involvement of *δ* and *θ* waves during creative thinking aligns with findings by Jung et al. (2013) on brainwave activity in creative tasks. The right brain’s increased δ wave activity supports findings that relaxation states enhance creative cognition (Arden et al., 2010a, 2010b). The involvement of both hemispheres suggests that creativity involves both analytical and imaginative processes (Dietrich, 2004). The current study’s neurophysiological findings are consistent with previous research, reinforcing the view that creativity and imagination arise from complex neural networks rather than isolated brain regions. However, the inclusion of amygdala activity suggests that emotional engagement may also play a role, which could be explored further in future research.

H2: The paired group t-test t-values for motivation to creativity in the experimental and control groups are *p* < 0.05; and *p* < 0.05, respectively. Certain brain regions demonstrate elevated activity, mainly concentrated in the prefrontal cortex, temporal lobe, and parietal lobe. This suggests a significant disparity between the pre-test and post-test scores of creativities in both groups. The study shows that playing online games is positively correlated with improving creativity. These results are consistent with the findings of the scale survey (H2) and confirm the reliability and validity of the study, such as Table 2.

**Table 2:** Scales and EEG analyses comparisons.

Method						
Hypothesis	Method 1: scale analysis			Method 2: EEG analysis		Judgement
	SPSS: regressive analysis		SEM: correlation ship/estimates			
H1:	*E*	F_(1,10)_ = 19.636, p = 0.001**	Positive/0.77***	*E*	p*** *< 0*.001	Supported
	*C*	F_(1,23)_ = 85.602, p = 0.000***		*C*	p* < 0.05	
H2:	*E*	F_(1,10)_ = 56.867, p = 0.000***	Positive/0.45***	*E*	p* < 0.05	Supported
	*C*	F_(1,23)_ = 56.630, p = 0.000***		*C*	p* < 0.05	
H3:	-		Positive/1.02	-		Supported

1. “- “: Not available. 2.*indicates *p* < 0.05; ** indicates *p* < 0.01; *** indicates *p* < 0.001. 3. E: Experimental group (Play Genshin Impact); C: Control group (Play Roblox). Sources: Drawn by the author.

Moreover, significant differences were observed in the pre-test and post-test brainwave measurements of a group of participants, particularly in areas concentrated in the Prefrontal Cortex (a), Parietal Lobe (b), Occipital Lobe (c), and adjacent to the Amygdala (d). Notably, the right hemisphere showed significant differences in δ waves, which may increase during relaxation associated with creative and imaginative activities. The *δ* brain waves, with a frequency range of 0.5 to 4 Hz, are typically linked to deep sleep, meditation, and relaxation. Research suggests that enhanced delta wave activity may be connected to unconscious creative processes and deep-level thinking, facilitating information integration and the generation of new ideas (Baird et al., 2012).

In addition, significant differences in *θ* waves were noted, which may increase during creative thinking and imagination. Theta brain waves, ranging from 4 to 8 Hz, are often associated with light sleep, meditation, and relaxation. Increased theta activity is commonly related to heightened creative thinking, intuition, and inner insight. Many creative activities and spontaneous thoughts are more pronounced under the influence of this brain wave activity, enhancing the ability to form associations between different concepts and facilitating creative problem-solving (Vartanian and Colombo, 2014). Furthermore, significant differences in *δ* waves (*p* < 0.05) were also observed in the left hemisphere. These findings relate generally to brain regions associated with stimulating imagination and creativity, as illustrated in Figure 4.

Current research shows that the brain regions involved in stimulating imagination and creativity comprise a complex and variable process, and there is no definite and consistent conclusion that points to specific brain areas directly causing these abilities. However, some studies indicate that the neural networks involved in creative thinking and imagination include the following brain regions (Abraham et al., 2012; Arden et al., 2010a, 2010b; Ellamil et al., 2012a, 2012b; Fink and Benedek, 2014):

1. Prefrontal Cortex (also see “a” in Figure 4): The prefrontal cortex, located at the front of the brain, is crucial for higher-order cognitive functions such as problem-solving, decision-making, and planning. According to Miller and Cohen (2001), increased activity in this region is linked with creative thinking and the ability to integrate complex information.
2. Parietal Lobe (also see “b” in Figure 4): The parietal lobe is involved in spatial perception and orientation, which are essential for visual and artistic creativity (Ellamil et al., 2012a, 2012b).
3. Occipital Lobe (also see “c” in Figure 4): The occipital lobe plays a crucial role in visual processing. Since creative thinking and imagination often involve visual elements, this region is integral to these processes (Fink and Benedek, 2014).
4. Amygdala (also see “d” in Figure 4): The amygdala is involved in emotional processing, and creativity and imagination may be closely related to emotional drives (Arden, 2010).

Research indicates that the brain regions involved in stimulating imagination and creativity comprise a complex and variable process. Currently, there is no clear and consistent conclusion that specific brain regions directly cause these abilities. However, studies suggest that the neural networks involved in creative thinking and imagination include the prefrontal cortex, parietal lobe, occipital lobe, and amygdala. These regions are responsible for higher-order cognitive functions, spatial perception, visual processing, and emotional processing, respectively, each playing a crucial role in creative thinking and imagination. Therefore, the connections between these brain regions and creativity should not be overlooked. Future research should continue to explore these complex neural mechanisms.

### EEG results

#### Multifaceted neural interpretation

Although the current discussion addresses the effects of δ (delta) and *θ* (theta) brainwaves, it could be further enriched by emphasizing the nonlinear characteristics of these waves and their association with the fluctuating nature of creative ideation. This aligns with Martindale’s (1999) description of creativity as a cognitive state that is not constant but marked by sudden bursts of insight and mental activity.

#### The role of emotion in motivation

While the involvement of the amygdala in emotional processing is mentioned, the link between emotion and motivation is not yet explicitly stated. It is worth highlighting that emotion intensity, often driven by motivational states, modulates activation in brain regions responsible for creativity. This reflects a central theme in recent research on emotion–cognition integration (Pessoa, 2008).

#### Cross-regional neural network integration

To deepen the neural analysis, the discussion could incorporate the interplay between the default mode network (DMN) and the executive control network (ECN), which are believed to coordinate during creative thought. Creativity, therefore, does not result from isolated brain regions, but from the collaborative activation across distributed networks—a notion supported by Beaty et al.'s (2015) meta-analysis.

This study not only confirms the positive predictive effects of motivation on imagination and creativity, but also enhances the reliability and validity of the psychological measures through neurophysiological evidence. Future research could explore how different types of motivation (e.g., intrinsic vs. extrinsic) affect creative performance, and examine moderating variables such as gender, age, or cultural background. Furthermore, the findings have valuable practical implications in fields such as educational technology, creative industries, and digital psychological interventions, warranting broader application and further investigation.

## Conclusion

This study provides significant insights into the relationship between motivation, imagination, and creativity within the context of digital gaming. Using both Structural Equation Modelling (SEM) and Electroencephalography (EEG) analysis, the research thoroughly examines how cognitive and affective processes interact and contribute to creative behavior in gaming environments. The findings demonstrate both direct and indirect relationships among motivation, imagination, and creativity, while also revealing neural activity that supports these psychological mechanisms. These results add to the growing body of literature on creativity, offering new perspectives on its cognitive and neurological foundations.

A central finding of this study is the mediating role of imagination between motivation and creativity. Specifically, the results suggest that intrinsic motivation alone does not directly lead to creative outcomes; rather, imagination acts as a vital intermediary. This finding highlights the importance of creating environments that foster imaginative thinking, as motivation alone may not be sufficient to promote creativity. For game designers and educators, this indicates that designing immersive and engaging experiences that actively stimulate imaginative processes is crucial for encouraging creativity. Practically, features such as nonlinear narratives, customizable worlds, and emotionally engaging gameplay may play a key role in promoting imaginative thinking and, in turn, creative problem-solving.

The EEG data further support these findings, revealing increased activation in brain regions associated with creative cognition and emotional processing, including the prefrontal cortex, parietal lobe, occipital lobe, and amygdala. These neural patterns align with the theoretical understanding that creativity arises from the dynamic interaction between cognitive control and spontaneous, free-flowing thought. The results are consistent with the cognitive flexibility framework, suggesting that creativity is facilitated by the brain’s ability to switch between divergent and convergent thinking. This interplay between focused attention and imagination is clearly activated during game-based engagement, providing additional evidence for the role of imagination in creative processes.

From an educational perspective, the findings underscore the potential of game-based learning environments in fostering creativity. Specifically, the study suggests that promoting intrinsic motivation, autonomy, and exploratory learning can help students develop their imaginative and creative capacities. Educators could benefit from integrating game-inspired pedagogies—such as choice-based tasks, scenario-driven learning, and real-time feedback—into their teaching practices. These strategies can activate the motivational and imaginative pathways critical to fostering creativity, particularly for younger learners still developing these cognitive abilities.

The implications of this research are particularly relevant given the increasing presence of digital games in the lives of children and young adults. As online gaming continues to be a dominant form of recreation and learning, understanding how these environments influence cognitive and creative development is crucial. This study suggests that, when thoughtfully designed, games can serve as powerful tools for enhancing creative thinking, offering more than just entertainment value.

Looking ahead, future research should aim to replicate and extend these findings by exploring different age groups and cultural contexts. It will be essential to determine whether the relationships between motivation, imagination, and creativity remain consistent across diverse populations. Furthermore, longitudinal studies could provide valuable insights into the long-term effects of gaming on creativity, examining whether sustained engagement with game environments leads to lasting improvements in creative problem-solving skills.

In conclusion, this study makes a meaningful contribution to the understanding of creativity as a multifaceted construct shaped by both internal and external factors. By integrating psychological theory with neurophysiological evidence, it presents a comprehensive model of how creativity is activated in gaming contexts and how these processes can be intentionally cultivated through design and pedagogy. The findings reinforce the role of imagination as a cognitive bridge between motivation and creativity and offer practical recommendations for educators, designers, and policymakers seeking to foster creative capabilities in educational and digital environments.

### Limitations and future directions

This study investigates the influence of motivation and imagination on creativity, using a sample of young adult gamers primarily from higher education settings. Given the specificity of the participant group, the findings should be interpreted with caution when generalizing to broader populations, such as children, adolescents, or working professionals. Additionally, the selected gaming platforms—Genshin Impact and Roblox—represent particular types of digital play experiences and may not fully capture the diversity of gaming environments. Thus, the conclusions of this study are best viewed as exploratory within a defined demographic and genre context.

The research was conducted in two phases: an initial online survey, followed by a laboratory-based experiment involving electroencephalogram (EEG) monitoring during gameplay. While this mixed-method approach provides valuable insights into the behavioral and neural processes underlying creativity, certain limitations remain. Specifically, it was not possible to control for participants’ gaming or online activities outside the experimental sessions, which may have introduced variability into the data. The relatively small sample size and lack of strict control over gameplay duration may also affect the reliability and generalizability of the results.

Despite these limitations, the study yields meaningful findings. Motivation and imagination were found to be positively associated with creativity, and specific brain regions were identified as being involved in creative processing. However, further research is needed to establish causal relationships. Future studies should consider expanding the demographic scope to include younger learners, particularly K–12 students, to examine developmental differences in cognitive and motivational patterns. Moreover, exploring how cultural values and individual motivational differences influence creativity would offer valuable insights. Longitudinal designs, cross-cultural samples, and task-specific neuroimaging methods could further enhance our understanding of how motivation and imagination evolve into creative performance over time and across contexts.

In sum, this study highlights the central role of motivation and imagination in creative development. The findings carry both theoretical and practical implications, offering a framework for understanding the cognitive and neural mechanisms that underlie creative expression in digital gaming environments. These insights may inform the design of game-based learning tools and foster creative engagement, ultimately unlocking the creative potential of individuals in educational and interactive contexts.

## Data Availability

The original contributions presented in the study are included in the article/Supplementary material, further inquiries can be directed to the corresponding author.

## References

[ref1] Abraham A. Beudt S. Ott D. V. M. von Cramon D. Y. (2012). Creative cognition and the brain: dissociations between frontal, parietal-temporal and basal ganglia groups. Brain Res. 1482, 55–70. doi: 10.1016/j.brainres.2012.09.007, PMID: 22982590

[ref2] Acar O. A. van den Ende J. (2016). Knowledge distance, cognitive–search processes, and creativity: the making of winning solutions in science contests. Psychol. Sci. 27, 692–699. doi: 10.1177/0956797616634665, PMID: 27016241 PMC4873726

[ref3] Adachi P. J. C. Willoughby T. (2013). The effect of video game experience on the development of creativity. Comput. Hum. Behav. 29, 588–595. doi: 10.1016/j.chb.2012.10.020

[ref4] Almeida L. S. Prieto L. P. Ferrando M. Oliveira E. Ferrándiz C. (2008). Torrance test of creative thinking: the question of its construct validity. Think. Skills Creat. 3, 53–58. doi: 10.1016/j.tsc.2008.03.003, PMID: 40330846

[ref5] Amabile T. M. (1983). The social psychology of creativity: A componential conceptualization. J. Person. Soc. Psychol. 45, 357–376. doi: 10.1037/0022-3514.45.2.357

[ref6] Amabile T. M. (1996). “Creativity.” In Blackwell Encyclopedic Dictionary of Organizational Behavior. ed. N. Nicholson (Cambridge, MA, Harvard Business School: Blackwell Publishers).

[ref7] Anderson J. Rainie L. (2012). The future of big data. Pew Research Center. Washington, DC: Pew Research Center.

[ref8] Arden R. (2010). “The neurobiology of creativity: a cognitive neuroscience perspective” in Handbook of the Neuroscience of Creativity, vol. 103, 103–114.

[ref9] Arden R. Chavez R. S. Grazioplene R. Jung R. E. (2010a). Neuroimaging creativity: a psychometric view. Behav. Brain Res. 214, 143–156. doi: 10.1016/j.bbr.2010.05.015 20488210

[ref10] Arden R. Chavez R. S. Grazioplene R. Jung R. E. (2010b). The creative brain and its hemispheric specialization. Psychol. Bull. 136, 557–574. doi: 10.1037/a0019740

[ref11] Aziz-Zadeh L. Liew S. L. Dandekar F. (2013). Exploring the neural correlates of visual creativity. Soc. Cogn. Affect. Neurosci. 8, 475–480. doi: 10.1093/scan/nss021, PMID: 22349801 PMC3624959

[ref12] Baird A. A. Wagner A. D. Damaraju E. (2012). The role of the default mode network in creativity. Front. Hum. Neurosci. 6:56. doi: 10.3389/fnhum.2012.00056, PMID: 22438841 PMC3305887

[ref59] Barbot B. Besançon M. Lubart T. (2016). The generality-specificity of creativity: Exploring the structure of creative potential with EPoC. Learning and Individual Differences, 52, 178–187. doi: 10.1016/j.lindif.2016.10.004

[ref13] Beattie D. K. (2000). Creativity in art: the feasibility of assessing current conceptions in the school context. Assess. Educ. 7, 176–192. doi: 10.1080/713613328

[ref14] Beaty R. E. Benedek M. Kaufman S. B. Silvia P. J. (2015). Default and executive network coupling supports creative idea production. Sci. Rep. 5:10964. doi: 10.1038/srep10964, PMID: 26084037 PMC4472024

[ref15] Blake E. Barnes R. (2023). The best sandbox games on Roblox (2024) unleash your creativity. Available online at: https://www.compsmag.com/best/sandbox-games-on-roblox/

[ref16] Blanco-Herrera J. A. Gentile D. A. Rokkum J. N. (2019). Video games can increase creativity, but with caveats. Creat. Res. J. 31, 119–131. doi: 10.1080/10400419.2019.1594524

[ref17] Byrne B. M. (2016). Structural equation modeling with AMOS: Basic concepts, applications, and programming. 3rd Edn. New York, NY: Routledge.

[ref18] Chelazzi L. Perlato A. Santandrea E. D'Ardenne K. (2013). Rewards teach visual selective attention. Vis. Res. 85, 58–72. doi: 10.1016/j.visres.2013.04.012 23262054

[ref19] Cheng Y. H. (2019). The mediating effects of motivation for playing Pokémon go on internet gaming disorder and well-being. Am. J. Fam. Ther. 47, 19–36. doi: 10.1080/01926187.2019.1583614

[ref20] Cheng Y. H. (2021). Effects of playing online games on imagination. Think. Skills Creat. 41:100924. doi: 10.1016/j.tsc.2021.100924, PMID: 40335399

[ref22] Colello S. M. G. (2007). Imagination in children’s writing: how high can fiction fly? Available online at: http://www.hottopos.com/notand14/silvia.pdf

[ref23] Csikszentmihalyi M. (1990). Flow: The psychology of optimal experience. NewYork: Harper & Row.

[ref24] Csikszentmihalyi M. (1996). Creativity: flow and the psychology of discovery and invention. New York: Harper Perennial.

[ref25] Csikszentmihalyi M. (1999). “Implications of a systems perspective for the study of creativity” in Handbook of creativity. ed. Sternberg R. J. (Cambridge: Cambridge University Press).

[ref26] Deci E. L. Ryan R. M. (1985). Intrinsic motivation and self-determination in human behavior. New York: Springer Science and Business Media.

[ref27] Deci E. L. Ryan R. M. (2000). The "what" and "why" of goal pursuits: human needs and the self-determination of behavior. Psychol. Inq. 11, 227–268. doi: 10.1207/S15327965PLI1104_01

[ref28] Deterding S. (2015). The‘end’of gamification: a critique of the effectiveness of gamification for game design. In Proceedings of the 2015 Annual GamiFIN Conference. 207–217

[ref29] Di Bello F. Giamundo M. Brunamonti E. Cirillo R. Ferraina S. (2019). The puzzling relationship between attention and motivation: do Motor biases matter? Neuroscience 406, 150–158. doi: 10.1016/j.neuroscience.2019.03.011, PMID: 30876984

[ref30] Dietrich A. (2004). The cognitive neuroscience of creativity. Psychon. Bull. Rev. 11, 1011–1026. doi: 10.3758/BF03196731, PMID: 15875970

[ref31] Ellamil M. Dobson C. Beeman M. Christoff K. (2012a). Evaluative and generative modes of thought during the creative process. NuroImage 59, 1783–1794. doi: 10.1016/j.neuroimage.2011.08.008 21854855

[ref32] Ellamil M. Dobson C. Beeman M. Christoff K. (2012b). Evaluating the role of imagination in creative cognition. Nuropsychologia 50, 329–338. doi: 10.1016/j.neuropsychologia.2011.12.019

[ref33] Engelmann J. B. Pessoa L. (2007). Motivation sharpens exogenous spatial attention. Cogn. Affect. Behav. Neurosci. 7, 240–249. doi: 10.3758/CABN.7.3.240 17683222

[ref34] Fink A. Benedek M. (2014). EEG alpha power and creative ideation. Neurosci. Biobehav. Rev. 44, 111–123. doi: 10.1016/j.neubiorev.2012.12.002, PMID: 23246442 PMC4020761

[ref35] Fink A. Graif B. Neubauer A. C. (2014). The creative brain: investigating the neural basis of creativity. Hum. Brain Mapp. 35, 1981–1996. doi: 10.1002/hbm.22307, PMID: 23861343 PMC3895106

[ref36] Fornell C. Larcker D. (1981). Evaluating structural equation models with unobservable variables and measurement error. J. Mark. Res. 18, 39–50. doi: 10.1177/002224378101800104

[ref37] Gee J. (2003). What video games have to teach us about learning and literacy. Technol. Pedagogy Educ. 1:20. doi: 10.1145/950566.950595, PMID: 39076787

[ref38] Gee J. P. (2007). What video games have to teach us about learning and literacy. Comput. Hum. Behav. 22, 72–86. doi: 10.1016/j.chb.2005.05.014

[ref39] Gentile D. A. Anderson C. A. Yukawa S. Ihori N. Saleem M. Lim K. M. (2014). Video games, learning, and imagination: a cognitive approach. Dev. Psychol. 50, 123–135. doi: 10.1037/a0033277

[ref40] Green C. S. Bavelier D. (2003). Action video game modifies visual selective attention. Nature 423, 534–537. doi: 10.1038/nature01647, PMID: 12774121

[ref41] Groyecka A. Gajda A. Jankowska D. M. Sorokowski P. Karwowski M. (2020). On the benefits of thinking creatively: why does creativity training strengthen intercultural sensitivity among children. Think. Skills Creat. 37:100693. doi: 10.1016/j.tsc.2020.100693

[ref1001] Guilford J. P. (1967). The nature of human intelligence. New York, NY: McGraw-Hill.

[ref42] Hair J. F. Black W. C. Babin B. J. Anderson R. E. (2014). Multivariate data analysis. 7th Edn. Upper Saddle River, NJ: Pearson.

[ref43] Hall J. Herodotou C. Iacovides I. (2022). Measuring player creativity in digital entertainment games using the creativity in gaming scale. Chapter 12 in book title: Open world Learning1st Ed, pages, vol. 14. London: Routledge, eBook.

[ref44] Hayes M. T. Sameshima P. Watson F. (2017). Imagination as method. Int J Qual Methods 14, 36–52. doi: 10.1177/160940691701400104

[ref45] Hodent C. (eds.) (2017a). The Gamer’s brain: how neuroscience and UX can impact video game design. Boca Raton, FL: CRC Press, Taylor & Francis Group.

[ref46] Hodent C. (2017b). “Designing games for learning: creating the conditions for success” in The psychology of learning and motivation, vol. 67 (Elsevier), 1–23.

[ref47] Hsu Y. (2019). Advanced understanding of imagination as the mediator between five-factor model and creativity. J. Psychol. 153, 307–326. doi: 10.1080/00223980.2018.1521365 30507357

[ref48] Hsu Y. Liang C. Chang C. C. (2014). The mediating effects of generative cognition on imagination stimulation. Innov. Educ. Teach. Int. 51, 544–555. doi: 10.1080/14703297.2013.796715

[ref49] Johnson-Laird P. N. (1983). Mental models: Towards a cognitive science of language, inference, and consciousness. Cambridge, MA: Harvard University Press.

[ref1002] Jung R. E. Mead B. S. Carrasco J. Flores R. A. (2013). The structure of creative cognition in the human brain. Front. Hum. Neurosci. 7, 330. doi: 10.3389/fnhum.2013.00330 23847503 PMC3703539

[ref50] Kafai Y. B. Burke Q. (2015). Connected gaming: What making video games can teach us about learning and literacy. Cambridge, MA: MIT Press.

[ref51] Karwowski M. Soszynski M. (2008). How to develop creative imagination? Assumptions, aims and effectiveness of role play training in creativity (RPTC). Thinking Skills and Creativity, 3, 163–171. doi: 10.1016/j.tsc.2008.07.001

[ref52] Kiili K. (2005). Digital game-based learning: towards an experiential gaming model. Internet High. Educ. 8, 13–24. doi: 10.1016/j.iheduc.2004.12.001

[ref53] Kline R. B. (2016). Principles and practice of structural equation modeling. 4th Edn. New York, NY: Guilford Press.

[ref54] Korgaonkar P. Wolin L. (1999). A multivariate analysis of web usage. J. Advert. Res., 39, 53–68. Available online at: https://www.researchgate.net/publication/243782178_A_Multivariate_Analysis_of_Web_Usage

[ref55] Liang M. Chen J. (2013). Arylamine organic dyes for dye-sensitized solar cells. Chemical Society Reviews, 42, 3453–3488. doi: 10.1039/C3CS35372A 23396530

[ref56] Liang C. Y. Chia T. L. (2014). Reliability, validity, and factor structure of the imaginative capability scale. Creat. Res. J. 26, 106–114. doi: 10.1080/10400419.2014.873671

[ref57] Liang S. Zheng D. Standley D. M. Guo H. Zhang C. (2013). A novel function prediction approach using protein overlap networks. BMC Syst Biol. 7:61. doi: 10.1186/1752-0509-7-61 23866986 PMC3720179

[ref21] Lin H. H. Chuang J. H. Liu T. L. (2011). “Regularized background adaptation: a novel learning rate control scheme for Gaussian mixture modeling,” in IEEE Transactions on Image Processing 20, 822–836. doi: 10.1109/TIP.2010.2075938 20840901

[ref58] Lin W. S. Hsu Y. Liang C. (2014). The mediator effects of conceiving imagination on academic performance of design students. Int. J. Technol. Des. Educ. 24, 73–89. doi: 10.1007/s10798-013-9244-x

[ref60] Martindale C. (1999). “Biological bases of creativity” in Handbook of creativity. ed. Sternberg R. J. (Cambridge, UK: Cambridge University Press), 137–152.

[ref61] McCrae R. R. Costa P. T. Jr. (1997). “Personality trait structure as a human universal.” In Handbook of Personality Psychology. eds. R. Hogan, J. Johnson, and S. Briggs (San Diego, CA: Academic Press). 10.1037//0003-066x.52.5.509 9145021

[ref62] McGonigal J. (2016). 10 online games with a social purpose. Available online at: https://blog.ted.com/10-online-games-with-a-social-purpose/

[ref64] miHoYo. (2024). Genshin impact. Available online at: https://genshin.mihoyo.com/

[ref65] Miller E. K. Cohen J. D. (2001). An integrative theory of prefrontal cortex function. Annu. Rev. Neurosci. 24, 167–202. doi: 10.1146/annurev.neuro.24.120501.061356 11283309

[ref66] Nacke L. Lindley T. Kukka A. (2014). The importance of immersion in video gaming. In Proceedings of the 2014 annual conference on human factors in computing systems (pp. 2967–2976).

[ref68] NuroStat Tutorial and Manual Version 2.0. Available online at: www.appliedneuroscience.com

[ref69] Ott M. Pozzi F. (2012). Digital games as creativity enablers for children. Behav. Inform. Technol. 31, 1011–1019. doi: 10.1080/0144929X.2010.526148

[ref70] Park S. Y. Sun M. K. Roh S. Soh M. A. Lee S. H. Kim H.. (2016). The effects of a virtual reality treatment program for online gaming addiction. Comput. Methods Prog. Biomed. 129, 99–108. doi: 10.1016/j.cmpb.2016.01.015, PMID: 26860055

[ref71] Pessoa L. (2008). On the relationship between emotion and cognition. Nat. Rev. Neurosci. 9, 148–158. doi: 10.1038/nrn2317, PMID: 18209732

[ref72] Play Tracker (2020). Genshin impact on PlayStation (PS4). Available online at: https://playtracker.net/insight/game/63068

[ref73] Plucker J. A. Beghetto R. A. Dow G. T. (2004). Why isn't creativity more important to educational psychologists? Potentials, pitfalls, and future directions in creativity research. Educ. Psychol. 39, 83–96. doi: 10.1207/s15326985ep3902_1

[ref74] Pontes H. M. Demetrovics K. Z. Griffiths M. D. (2014). The conceptualization and measurement of DSM-5 internet gaming disorder: the development of the IGD-20 test. PLoS One 9, 110–137. doi: 10.1371/journal.pone.0110137, PMID: 25313515 PMC4196957

[ref75] Przybylski A. K. Rigby C. S. Ryan R. M. (2010). The motivating role of games in cognitive engagement. Psychol. Sci. 21, 489–495. doi: 10.1177/095679761036267020424088

[ref76] Rafner J. Biskjær M. M. Zana B. Langsford S. Bergenholtz C. Rahimi S.. (2021). Digital games for creativity assessment: strengths, weaknesses and opportunities. Creat. Res. J. 34, 28–54. doi: 10.1080/10400419.2021.1971447, PMID: 40330611

[ref77] Roblox (2022). A year on ROBLOX: 2021 in data. Available online at: https://corp.roblox.com/newsroom/2022/01/year-roblox-2021-data

[ref78] Runco M. A. (2014). Creativity and video games: trends and implications. Creat. Res. J. 26, 171–178. doi: 10.1080/10400419.2014.901042

[ref79] Runco M. A. Acar S. (2012). Divergent thinking as an indicator of creative potential. Creat. Res. J. 24, 66–75. doi: 10.1080/10400419.2012.652929

[ref80] Ryan R. M. Deci E. L. (2000). Self-determination theory and the facilitation of intrinsic motivation, social development, and well-being. Am. Psychol. 55, 68–78. doi: 10.1037/0003-066X.55.1.68, PMID: 11392867

[ref81] Ryan R. M. Deci E. L. (2020). Intrinsic and extrinsic motivation from a self-determination theory perspective: definitions, theory, practices, and future directions. Contemp. Educ. Psychol. 61:101860. doi: 10.1016/j.cedpsych.2020.101860

[ref82] Seligman M. Kaufman S. B. (2014). Advancing the science of imagination: toward an ‘imagination quotient’. Request for proposals: an international grants competition for research and intervention projects on the measurement and improvement of imagination. Available online at: http://www.imagination-institute.org

[ref83] Statista (2024a). Genshin impact. Available online at: https://www.statista.com/topics/10100/genshin-impact/

[ref84] Statista (2024b). Roblox—statistics and facts. Available online at: https://www.statista.com/topics/7594/roblox-corporation/#topicOverview

[ref85] Vartanian O. Colombo M. (2014). The neuroscience of creativity: theoretical approaches and empirical findings. Psychol. Bull. 122, 756–769. doi: 10.1037/a0033407, PMID: 24016014 PMC4047525

[ref86] Wang B. Ginns P. Mockler N. (2022). Sequencing tracing with imagination. Educ. Psychol. Rev. 34, 421–449. doi: 10.1007/s10648-021-09625-6

[ref87] Wang C. C. Ho H. C. Wu J. J. Cheng Y. Y. (2014). Development of the scientific imagination model: A concept-mapping perspective. Think. Skills Creat. 13, 106–119. doi: 10.1016/j.tsc.2014.04.001

[ref88] Winkelhofer G. (2006). Kreativ managen: Ein leitfaden für unternehmer, manager und projektleiter. Berlin/Heidelberg/New York: Springer Science and Business Media.

[ref90] Yee N. (2006). Motivations for play in online games. Cyberpsychol. Behav. 9, 772–775. doi: 10.1089/cpb.2006.9.772, PMID: 17201605

