# Supplementary material for: The impact of online games on creativity and the role of imagination
Source: Front Behav Neurosci. 2025 May 22;19:1561548. doi: 10.3389/fnbeh.2025.1561548 (PMC12137278; doi:10.3389/fnbeh.2025.1561548)
Supplement: Supplementary file 1 [file Supplementary_file_1.docx]

Appendix

I. Online Gaming Motivation Scale: Revised from Hodent (2017)

Implicit motivation and biological drives

1. I enjoy assuming the role of a leader in games.

2. I often strive to accomplish challenging tasks or goals in games.

3. I tend to form close connections or friendships with other players in games.

4. Achieving success in games gives me a strong sense of accomplishment.

Environmental-shaped motivation and learned drives

5. The reward system in games motivates me to repeatedly engage in specific tasks or behaviors.

6. I find games more engaging when the outcomes are uncertain.

7. I avoid certain behaviors in games out of fear of in-game penalties.

8. The rewards in games encourage me to participate more actively.

Intrinsic motivation and cognitive needs

9. Completing certain tasks in games enhances my sense of competence.

10. I feel satisfied when I can autonomously decide my actions in games.

11. When I focus on a particular goal in games, I lose track of time and become fully immersed.

12. I enjoy challenging my limits in games and mastering specific skills.

Personality and individual needs

13. My personality influences my behavior in games.

14. My personal interests affect the types of games I choose to play.

15. My gaming motivations resemble my real-life motivations.

16. My gaming style reflects my personality traits.

II. Imagination scale: Revised from Liang and Chia (2014)

Initiating imagination:

1. I can come up with unique ideas.

2. I can generate a wide range of ideas.

3. I can continuously come up with different ideas.

4. I can challenge the original ideas of others.

Conceptualizing imagination:

5. I can quickly identify the key points from a vast amount of information.

6. I can continuously come up with different ideas.

7. I can stay focused on a task until an idea takes shape.

8. I can carefully analyze contradictions in a problem.

Transforming imagination

9. I can express abstract concepts using examples from daily life.

10. I can explain unfamiliar concepts using examples familiar to others.

11. I can integrate others' opinions into my own ideas.

12. I can adapt similar ideas to different contexts.

III. Creative Ability Scale: Revised from Hall, Herodotou, and Iacovides (2022)

Transferability:

1. I have used what I learned from games in my work or workplace.

2. Gaming has influenced my attitude or behavior in other areas of life.

3. Playing games has given me a different perspective on everyday life.

4. Expressing creativity in games has given me new perspectives on problems and challenges in daily life.

5. Expressing creativity in games has developed my problem-solving and thinking abilities.

Appropriation:

6. I actively seek out and address gaps.

7. I enjoy using game mechanics in new and unexpected ways.

8. I try to find shortcuts in games.

9. Fixing minor glitches allows me to progress in games.

10. I enjoy testing the limits of what the game allows.

Problem solving:

11. I enjoy coming up with new strategies while playing games.

12. I like creating additional challenges for myself in games, such as increasing the difficulty.

13. I highly value the sense of achievement when overcoming difficult challenges in games.

14. I like experimenting with changing game parameters to innovate gameplay.

15. I enjoy games that allow me to try different styles.

Affective change:

16. The story in the game is important to me.

17. I enjoy games that make me question things.

18. I enjoy games that give me new perspectives on other cultures and societies.

19. I prefer games that allow me to choose my character's personality through dialogue.

Design affordances

20. Games that offer more freedom to players are more likely to involve creativity.

21. Games that allow free construction and creation are the most creative.

22. Games that provide opportunities for interaction with the environment help me be more creative while playing.
